# Auditory Cortex Maturation and Language Development in Children with Hearing Loss and Additional Disabilities

**DOI:** 10.3390/children10111813

**Published:** 2023-11-15

**Authors:** Satu Lamminmäki, Kayla Cormier, Hanna Davidson, Jim Grigsby, Anu Sharma

**Affiliations:** 1 Department of Speech Language and Hearing Sciences, University of Colorado Boulder, 2501 Kittredge Loop Dr. UCB 409, Boulder, CO 80309, USA; 2 Department of Otorhinolaryngology-Head and Neck Surgery, University of Helsinki and Helsinki University Hospital, P.O. Box 263, 00029 HUS, Helsinki, Finland; 3 Department of Psychology, University of Colorado Denver, Denver, CO 80217, USA

**Keywords:** hearing loss, disability, language, electroencephalogram, EEG, cortical, auditory, evoked potential, CAEP, P1 biomarker

## Abstract

A significant portion of hearing-impaired children have additional disabilities, but data about the maturation of their auditory cortex are scarce. In these children, behavioral tests are often unreliable, and objective tests are needed for diagnostics and follow-up. This study aimed to explore auditory cortical maturation and language development, and the usability of an objective electroencephalogram-based biomarker in children with multiple disabilities. In 65 hearing aid and cochlear implant users (36 females; 36 with multiple disabilities; 44.3 ± 18.5 months of age, mean ± SD), auditory processing was examined using the P1 cortical auditory evoked response biomarker, and language development with the Preschool Language Scales 5th edition (PLS-5). During the study, all of the children received intensive extra language therapy for six months. No significant differences were found between the groups in P1 latency development, the proportion of abnormal P1 latencies, or the number of children whose P1 latencies changed from abnormal to normal during the study. The PLS-5 total language scores, auditory comprehension scores, or expressive communication scores did not differ between groups either. The P1 latencies showed meaningful negative correlations with the language scores. The results suggest that auditory cortex development is similar in hearing-impaired children with/without additional disabilities, and the P1 biomarker is a feasible tool to evaluate central auditory maturation in children with multiple disabilities.

## 1. Introduction

Children with hearing loss frequently have additional disabilities, either because of genetic mutations and/or environmental factors. The overall incidence of congenital severe hearing loss is about 1 in 1000 births [1], and about half of all congenital hearing loss cases have genetic bases; of these, about 20% are syndromic [2]. More than 650 hereditary syndromic hearing loss entities are known [3,4], most of them having either autosomal recessive (e.g., Usher, Pendred, and Jervell and Lange–Nielsen syndromes) or autosomal dominant inheritance (e.g., Waardenburg, CHARGE, Treacher Collins, and brachio-oto-renal syndromes). In addition, X-linked inheritance (e.g., Alport syndrome) and rarely mitochondrial inheritance (e.g., MELAS syndrome) can occur. In addition to genetic syndromes, a variety of acquired conditions, such as infections (e.g., cytomegalovirus, rubella, toxoplasmosis, meningitis), prematurity, infant hypoxia, hyperbilirubinemia, and neonatal intensive care, may lead to both hearing loss and additional disabilities of varying severity. Hearing-impaired children can also have comorbidities which have no association with the hearing loss. Altogether, approximately 20–40% of deaf or hard of hearing children have an additional disability [5,6,7,8].

The evaluation of hearing—both without and with hearing devices—can be challenging in children, and especially in children with multiple disabilities. Newborn hearing screening is based on easily measurable otoacoustic emissions and automatic ABR, and these methods can also be used in older children if they sleep peacefully or cooperate well. However, the diagnosis of hearing loss requires more detailed evaluation. In newborns and young infants (recommended under 8 weeks of age [9], at most roughly 6 months of age), the degree of hearing loss can usually be objectively assessed with the auditory brainstem response (ABR) measurement during natural sleep. Frequency-specific estimates of hearing loss can be obtained, e.g., using tone pip/burst or narrow-band chirp ABR, or by combining click ABR with auditory steady-state response (ASSR) measurements [9,10,11]. Typically, additional disabilities cause no major problems in this evaluation if the child sleeps long enough for all of the measurements to be completed. Different brain diseases may affect the ABR morphology, but in most cases these small changes do not interfere with the assessment of hearing thresholds [12,13]. However, hearing loss can have a late onset or be progressive, resulting in the hearing evaluation being delayed. A reliable diagnosis of hearing loss can be obtained by pure tone audiogram roughly around the time the child is 5 years old [14]. In toddlers and non-cooperating older children, the diagnosis must be based on objective measures. In these children, the diagnostic threshold ABR normally requires sedation, because they do not sleep or wake up easily, disrupting the reliability of ABR measurement. Occasionally, sedation is also required in newborns and young infants if the data otherwise contain too much motion artifact. Unfortunately, anesthetic risks are often higher in children with multiple disabilities, and sedation may be contraindicated even for diagnostic purposes. The need for hearing evaluation does not end in the diagnosis of hearing loss, but the efficacy of hearing rehabilitation must be monitored regularly. Sometimes, even improvements in hearing may be seen during follow-up [15]. Typically, the follow-up of hearing-impaired children is largely based on different behavioral tests, such as visual reinforcement audiometry, speech perception, and tests for assessing language development, but these tests may be impossible to implement in children with multiple disabilities, or the results may be unreliable. Adequate assessment is crucial for early identification in all children who do not benefit sufficiently from conventional hearing aids, and for whom cochlear implantation is indicated.

In years past, children with severe to profound hearing loss and additional disabilities were often considered poor candidates for cochlear implants (CIs) [16]. As a result, these children have been excluded from many previous studies [17,18,19,20,21], and data are scarce on the maturation of the auditory system in children with hearing loss and additional disabilities. However, the indications for cochlear implantation have expanded, and the understanding of the importance of hearing in the presence of other (e.g., sensory or motor) handicaps has increased. Current CIs also contain a method that directly measures cochlear nerve stimulation thresholds, which can help in guiding the programming of CIs in children whose behavioral responses are unreliable. Currently, the majority of deaf children with additional disabilities receive CIs in many countries. However, children with multiple disabilities have shown variable clinical outcomes after CI use [22]. In some children, speech perception and language development are comparable to hearing-impaired children without other disabilities [5,23]. whereas some children with multiple disabilities benefit from CIs, but do so at a slower pace and to a lesser degree [5,23,24,25]. Taken together, an easy, objective, and non-invasive test is needed to identify of children whose hearing (with or without intervention) is not adequate for normal auditory maturation, and who should be considered candidates for CIs. Furthermore, monitoring the progress of hearing aid and/or CI treatment and accompanying auditory maturation is often difficult in children with multiple disabilities, and objective tests are warranted for reliable follow-up.

Cortical auditory evoked potentials (CAEP) are generated in the auditory cortex in response to acoustic stimuli, and can be recorded by an electroencephalogram (EEG) on the scalp. CAEPs consist of a series of positive and negative deflections, which undergo steady changes during development [26,27,28,29,30]. The latency of the P1 wave can be used as a biomarker to infer the maturity of the central auditory pathways and auditory cortex [17,18]. Since in normal hearing children P1 latencies decrease with age, the auditory cortical maturation of hearing-impaired children can be assessed by comparing individual P1 latencies to age-normative data [18]. In a study of 245 congenitally deaf children, P1 latencies normalized within 3–6 months after CI activation if the child was under 3.5 years of age at the time of implantation. However, children who had 7 or more years of auditory deprivation before implantation never developed normal P1 latencies [19].

Sharma et al. explored the clinical relevance of the EEG-based P1 biomarker in three case studies of hearing-impaired children with multiple disabilities—all of whom had unreliable behavioral test results. In a 23-month-old child with CHARGE syndrome (coloboma of the eye, heart defects, atresia of the choanae, restriction of growth and development, and ear abnormalities and deafness), the P1 latencies were normal after 18 months of hearing aid use, whereas in a child with Pallister–Killian syndrome (an abnormal extra chromosome 12p causing hypotonia, intellectual disability, unusual skin pigmentation, and distinctive facial features), the latencies were delayed due to insufficient hearing aid amplification. A 31-month-old child with ANSD (auditory neuropathy spectrum disorder) showed normal P1 latencies in the ear with a CI, and no congruent P1 waves in the non-implanted ear. The authors concluded that the combination of hearing loss and additional disabilities poses unique challenges for deciding upon appropriate intervention for these children, and the P1 biomarker can be useful in assessing central auditory maturation. However, because of the very few subjects, no correlations between the P1 latencies and language development were examined. Currently, language development is the best available behavioral tool to evaluate appropriate central auditory maturation. Also, questionnaires for parents and educators provide valuable information about auditory skills. In addition to medical decision making, it is important for parents of these children to receive informed advice regarding development of the central auditory system and language acquisition to make decisions regarding early intervention and management using devices such as hearing aids or CIs [5,18,19,20,21,31].

The main goal of this study was to explore if auditory cortical development and language acquisition are more delayed in hearing-impaired children with additional disabilities than in children who have only hearing loss. The second goal was to investigate whether P1 testing is clinically feasible in hearing-impaired children with a variety of multiple disabilities. To these ends, we compared the P1 biomarker latencies and performance on tests of language development in children with multiple disabilities and hearing loss to those with only hearing loss.

## 2. Materials and Methods

This study employed a cohort study design. The data were collected as part of a larger prospective NIH/NIDCD study (grant number: 1U01DC013529) examining aural rehabilitation (in person vs. telehealth) in hearing-impaired children. As a result of the secondary use of a part of the data of the prospective NIH/NIDCD study, the design of this study is considered retrospective.

### 2.1. Participants

A total of 65 hearing-impaired, English-speaking children between the ages of 13 months and 78 months (44.3 ± 18.5, mean ± SD) were included in this study. Informed consent was obtained from all guardians of the participants prior to data collection. All of the participants presented with mild to profound hearing loss, and were treated with either hearing aids or CIs. Children with unilateral hearing loss and purely conductive hearing loss were excluded from this study. One child in the control group had an inconsistent secondary conductive component due to middle ear involvement. Hearing loss diagnoses were carried out according to standard age-appropriate clinical practice following the American Academy of Audiology guidelines [32]. A total of 36 children had multiple disabilities (Supplementary Materials), and 29 control children had only hearing loss (i.e., no additional disabilities). The multiple disabilities group covered a wide spectrum of different additional disabilities frequently found in hearing-impaired children in everyday clinical practice. The additional disabilities varied from congenital (genetic disorders) to acquired conditions (e.g., cytomegalovirus), and some of them had the same etiological background as their hearing loss, while for others the relations between the hearing loss and the additional impairment(s) were unclear. All of the children had received some speech and language therapy prior to the study, and during the study they received intensive listening and spoken language therapy services for 6 months, either in person or via telehealth. There were 45 children (23 children with isolated hearing loss and 22 children with multiple disabilities) who completed the whole test battery that included EEG and language measures, both during the first visit and at the follow-up visit. A power analysis with the power (1 − β) set at 0.80 and the α equal to 0.05 (two-tailed) indicated that a sample size of 39 children would be sufficient to detect a small effect size (d = 0.2). All 65 children participated in at least one language assessment, and 52 children participated in at least one CAEP measurement.

Table 1 summarizes the background information of all of the subjects (36 children with multiple disabilities and 29 children with isolated hearing loss), as well as the subjects with complete datasets (22 children with multiple disabilities and 23 children with isolated hearing loss). The groups showed no significant differences with reference to age (t(63) = 1.46, *p* = 0.15), gender (z(1) < 0.001, *p* = 1.00), proportion of hearing aid-only users, or proportion of CI users, including unilateral CI users with and without a hearing aid as well as bilateral CI users (z(1) = 0.17, *p* = 0.68). Additionally, there were no differences between the groups with regard to the age at which CI users obtained their first implant (t(39) = −0.92, *p* = 0.36). No differences existed between the groups on the mode of speech therapy delivery (z(1) < 0.001, *p* = 1.00) or in the length of therapy (t(41.22) = 1.03, *p* = 0.31). The results were consistent across the groups with complete datasets.

### 2.2. Biomarker of Auditory Development

The P1 CAEP biomarker was recorded to assess auditory development. The data were collected at two time points. The baseline appointment occurred around the time of enrollment in the larger NIH/NIDCD study, and follow-up appointments occurred roughly 6–7 months after the baseline appointment. The CAEPs were recorded in response to the synthesized speech syllable /ba/. The duration of the stimulus was 90 ms. This same stimulus has been used in many of our previous studies [17,18,30]. The stimulus was delivered at 65 dB HL via two loudspeakers located at a 45° azimuth. Testing for the subjects occurred in an electromagnetically shielded sound booth where the child was seated on a parent’s lap in a comfortable chair. The child viewed a movie of their choice that was played with no sound on a TV monitor in front of them. The test session, including electrode application and CAEP recordings, lasted approximately an hour. For all testing, the subjects’ hearing aids or CIs were set at their normal settings.

The brain responses were measured with 5–8 electrodes, depending on if the child had a hearing aid or CI. Eight electrodes along the isopotential contours were utilized for children with CIs to minimize the CI artifact, as described using a previously published method [33]. The active electrode was located at Cz, and a ground electrode at Fpz. The cortical responses were recorded and analyzed using the Scan acquisition software (Compumedics Neuroscan). The raw data were sampled at 1000 Hz and filtered from 0.1 to 1000 Hz. The epochs contaminated by eye movements or other external artifacts (over ±100 µV) were rejected. At least 300 epochs were averaged online for each participant using a 100 ms pre-stimulus and a 600 ms post-stimulus time window. To ensure replicability of the responses, at least two runs were completed, and a grand average of two replicable responses was created to determine the P1 latencies. The P1 latencies were visually selected by trained audiologists. Children without a replicable P1 response were not included in analyses examining P1 latencies; however, these children were included in analyses when using the categorical abnormal P1 outcome. The change in P1 latency was obtained by subtracting the baseline latency from the follow-up latency. The change in P1 rate was computed by first normalizing raw P1 latencies by subtracting the upper, 95% confidence limit of the age-matched normative latencies from the raw P1 latency [18]. The change in normalized P1 latencies was then divided by the change in age from baseline to follow-up.

### 2.3. Language Assessment

The Preschool Language Scales 5th edition (PLS-5) is a comprehensive tool kit for play-based assessment of language development in children under 7 years of age. It can be used in person or via telehealth. The PLS-5 measures include scores for auditory comprehension (AC), expressive communication (EC), and a total language score, which is derived from the AC and EC scores. All of the raw scores are converted to age equivalent scores in months [34,35].

The PLS-5 was completed at baseline and at follow-up by speech language pathologists or teachers of the deaf/hard of hearing as part of a larger study. The baseline and follow-up measurements of language development and CAEPs occurred around the same time. Difference scores were created by subtracting the baseline age equivalent scores from the follow-up age equivalent scores. The changes in rate scores were computed by dividing the change in age equivalent scores by the change in age from baseline to follow-up.

### 2.4. Statistical Analysis

The statistical analysis was conducted with R (version 4.2.1) [36] and Rstudio, an integrated development environment for R [37]. The potentially confounding effects of missing data were first examined by determining the differences between participants with complete and incomplete datasets. Participants with incomplete datasets were significantly younger (*p* = 0.01). Therefore, multilevel model (MLM) analyses, completed with the lme4 package [38], were applied only to participants with complete datasets, while *t*-test comparisons utilized all participant data.

The applicability of statistical models was evaluated by examining the model residuals via quantile–quantile plots and histograms. Logistic MLM residuals revealed a heavy tailed distribution for models employing difference scores, and a bimodal distribution for models employing changes in P1 rates (change in age-adjusted scores/change in age). However, because the MLM is robust to violations of normality, a logistic MLM with a random intercept and a group binary dependent variable was applied to the complete datasets [39]. The changes in P1 cortical auditory latencies, expressive communication, and auditory comprehension were investigated, whereas the total language scores were excluded from the MLM, as the total scores are derivatives of the expressive communication and auditory comprehension outcomes. Prior to analysis, the cortical auditory measures were z-scored. A logistic MLM with a random intercept and a group binary dependent variable was also employed to examine the difference in change rate of the independent variables.

The Shapiro–Wilk test was completed to assess assumptions of normality. Due to a lack of normality across all variables, transformations were performed such that analyses were conducted with the inverse of the P1 latencies, the square root of the PLS-5 age equivalent scores at baseline, and the log of the PLS-5 age equivalent scores at follow-up. However, the P1 baseline latencies in the disability group as well as the P1 latencies at baseline collapsed across the groups, and continued to demonstrate evidence of a non-normal distribution. A non-normal distribution was also indicated for the transformed PLS-5 expressive communication score.

For the *t*-test comparisons, both parametric (*t*-tests) and non-parametric (Mann–Whitney tests) were performed. Non-parametric tests were used to ensure that the non-normally distributed data did not interfere with the results. Since the results were not different between the parametric and non-parametric tests, only the parametric test results are reported in this study, because parametric tests are commonly used in medical research [40,41,42] and are easier to interpret. Two-tailed independent sample *t*-tests were used to assess group differences between those with multiple disabilities and individuals with isolated hearing loss in the cortical auditory and language measures. *t*-tests were completed for baseline measures, follow-up measures, and the change in outcomes between these two time points. Unpaired *t*-test with unequal variances were used when appropriate, based upon a test of the variance. Two proportion z-tests were used to examine the proportion of children with abnormal P1 latencies in comparison to age-matched normative values for each group.

Linear regressions were examined between P1 latencies and all PLS-5 scores at baseline and follow-up for all children, regardless of group. Linear regressions were also performed based on categorization as an abnormal P1 latency and normal P1 latency. However, because only 11 children had normal P1 latencies at baseline, and only 9 children had abnormal P1 latency at follow-up, linear regressions for these statistically underpowered sub-groups were not calculated.

The *p*-values were adjusted to account for multiple comparisons using the Benjamini–Hochberg false discovery rate procedure to reduce the risk of type I errors [43]. Both the unadjusted and adjusted *p*-values are reported because in this study, the correlation coefficients are highly correlated with each other, and multiple comparison corrections are primarily aimed at independent, non-correlated simultaneous statistical tests.

## 3. Results

### 3.1. Individual Latencies of P1 Cortical Auditory Evoked Responses

**Figure 1:**
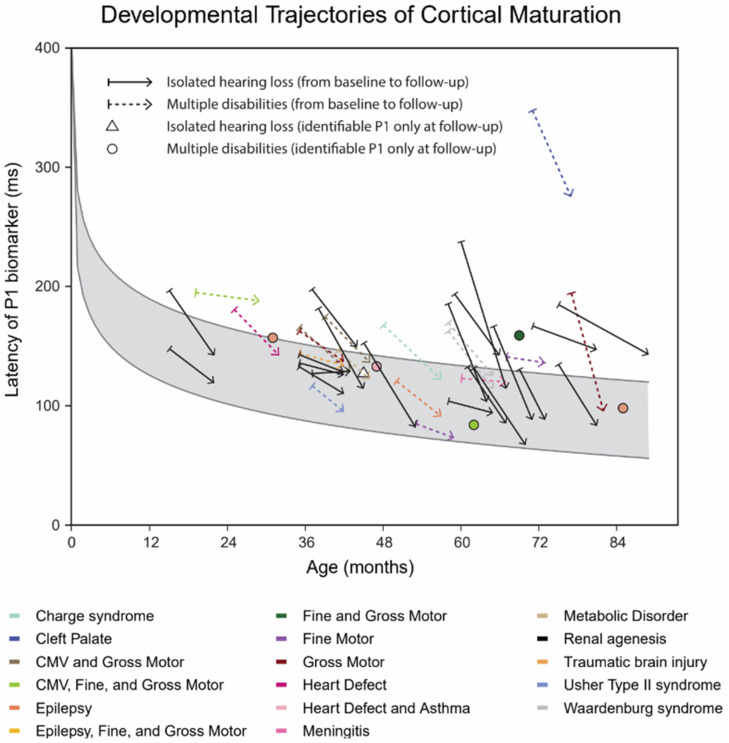
Individual CAEP P1 latency trajectories in children with complete datasets. Straight arrow lines show the improvement of the individual latencies from baseline (20 children with isolated hearing loss and 17 children with multiple disabilities) to follow-up (22 children with isolated hearing loss and 22 children with multiple disabilities): solid lines depict children with isolated hearing loss, and dashed colored lines depict children with multiple disabilities. Different disabilities are shown with different colors. Confidence intervals for age-matched normative values are indicated by the light gray areas. CMV = cytomegalovirus.

Figure 1 illustrates individual P1 latencies of 44 out of 45 children with complete datasets, plotted against chronological age. Three children with isolated hearing loss and five children with multiple disabilities had abnormal CAEP patterns at baseline, and no clearly identifiable P1 responses [18]. One child out of forty-five had no clear P1 response at baseline or at follow-up. At baseline, P1 latencies of 12 children with isolated hearing loss and of 12 children with multiple disabilities were abnormally late when compared to age-matched normative values (95% confidence interval), as outlined by the gray lines [18]. With the exception of one child, the P1 latency decreased between the baseline and the follow-up measurements (downward sloping lines). In one child with isolated hearing loss, the P1 latency showed a negligible 2 ms increase during follow-up.

### 3.2. Group Differences in P1 Latencies

Regarding the proportions of abnormal baseline P1 latencies, no significant differences existed between the groups (65% for children with isolated hearing loss vs. 77% for children with multiple disabilities; z(1) = 0.32, *p* = 0.57, adjusted *p* = 0.66). In addition, the proportion of children who had abnormal P1 latencies at baseline and normal P1 latencies at follow-up visits were similar in the group of multiple disabilities (23%) and in the children who had only hearing loss (17%) (z(1) = 0.006, *p* = 0.94, adjusted *p* = 0.99).

**Figure 2:**
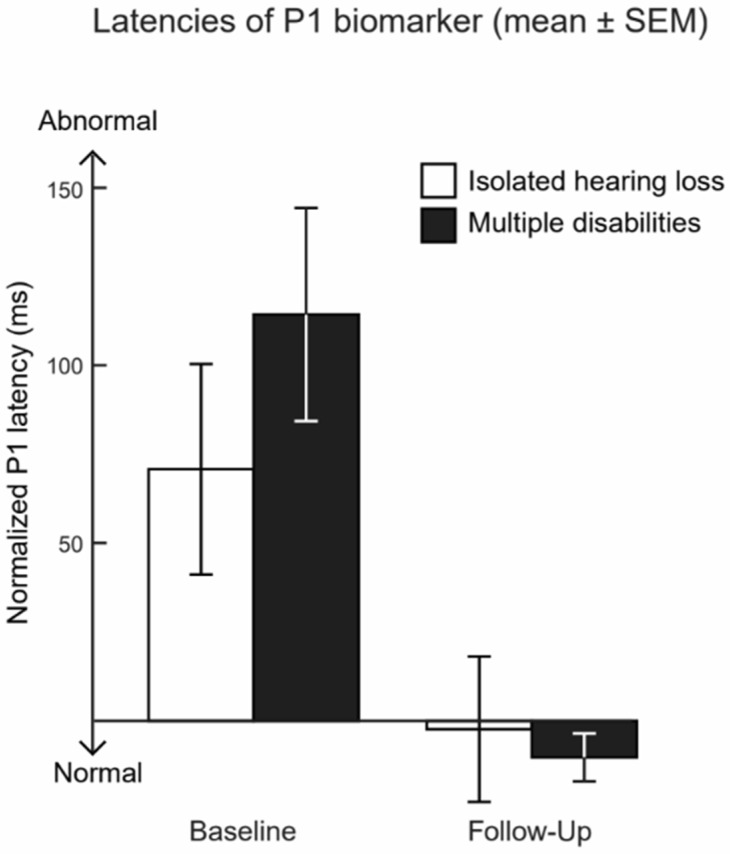
Average age-normalized CAEP P1 latencies at baseline and follow-up (mean ± SEM, in ms). Negative values indicate the raw P1 latencies within normal limits in comparison to age-matched normal hearing peers, and positive values indicate abnormally long P1 latencies. At baseline, 21 children with isolated hearing loss and 18 children with multiple disabilities had identifiable P1 responses; at follow-up, the numbers were 22 and 23, respectively.

Figure 2 shows the mean (± SEM) normalized P1 latencies of all children with identifiable P1 response at baseline (*n* = 37) and follow-up (*n* = 45). Positive values indicate abnormally long latencies, when compared with age-matched normative values, and negative values indicate the P1 latencies are within the upper 95% confidence limit of the normative values. For both groups, the mean of the age-normalized P1 latencies were abnormally long at baseline (mean ± SEM; 16.36 ± 6.57 for children who had only hearing loss and 18.39 ± 9.62 for children with multiple disabilities), and normal at follow-up (−23.55 ± 4.43 and −10.27 ± 7.02, respectively). In children who participated in both baseline and follow-up P1 measurements, the P1 latencies significantly changed from baseline to follow-up in each group (t(19) = 5.93, *p* < 0.001, adjusted *p* < 0.001 for children who had only hearing loss and t(16) = 3.67, *p* = 0.002, adjusted *p* = 0.02 for children with multiple disabilities, paired *t*-test). However, no significant differences in the P1 latencies at baseline (t(37) = −0.27, *p* = 0.79, adjusted *p* = 0.86) or follow-up (t(43) = 1.31, *p* = 0.20, adjusted *p* = 0.27) existed between the group of children with isolated hearing loss and the group of children with multiple disabilities. The change in P1 latencies from baseline to follow-up also demonstrated no significant differences (t(36) = 1.70, *p* = 0.10, adjusted *p* = 0.27) between the groups, being 46.80 ± 5.64 ms (mean ± SEM) for children with isolated hearing loss and 36.67 ± 4.69 ms for the children with multiple disabilities. The MLM analyses, limited to the children with the full datasets, yielded consistent results (95% [0.15, 1.19], *p* = 0.10, adjusted *p* = 0.26) after controlling for age at baseline, the change in age from baseline to follow-up, language outcomes, and allowing for a random intercept. Furthermore, examining the rate of change defined as the difference score divided by the change in age also demonstrated a lack of significant differences between the multiple disabilities group and the children with isolated hearing loss for cortical auditory measures (95% CI [0.24, 1.28], *p* = 0.17, adjusted *p* = 0.31).

### 3.3. Group Differences in Language Outcomes

**Figure 3:**
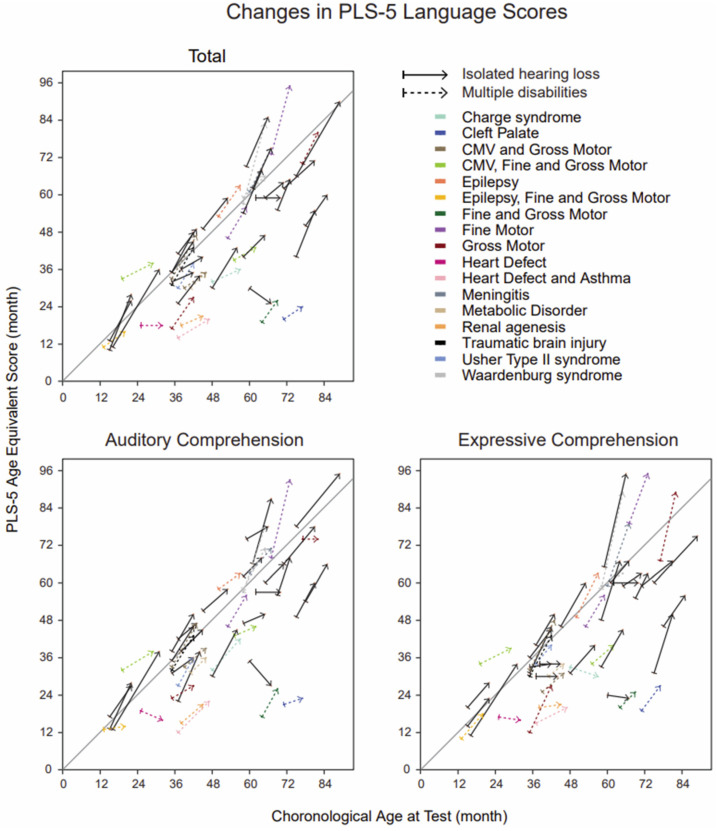
Individual PLS-5 score trajectories in children with complete datasets. Arrow lines demonstrate the improvements in individual age equivalent scores from baseline to follow-up (23 children with isolated hearing loss and 22 children with multiple disabilities): solid lines depict children with isolated hearing loss, and dashed colored lines depict children with multiple disabilities. Different disabilities are shown with different colors. The linear gray lines show the normal development trajectories. CMV = cytomegalovirus.

Figure 3 illustrates the individual PLS-5 total language scores, PLS-5 AC scores, and PLS-5 EC scores at baseline and after follow-up for those children who participated in both language assessments. During the follow-up, the age-equivalent total PLS-5 language scores improved for all of the children except for one child with isolated hearing loss (5 months decrease) and one child with hearing loss and a heart defect (1 month decrease). The same model, as described above with the P1 latencies, was applied to these children with complete datasets (*n =* 45). Similar to the findings of the P1 latencies, no significant differences existed between the multiple disabilities group and the group of isolated hearing loss in the change in PLS-5 EC scores (95% CI [0.88, 1.06], *p* = 0.48, adjusted *p* = 0.59), or change in PLS-5 AC scores (95% CI [0.93, 1.19], *p* = 0.46, adjusted *p* = 0.59); the odds of obtaining the average scores on these language measures were approximately equal between groups. The rate of change defined as the difference score divided by the change in age also revealed a lack of significant differences between the multiple disabilities group and the isolating hearing loss group for language measures (EC: 95% CI [0.37, 1.31], *p* = 0.27, adjusted *p* = 0.39; AC: 95% CI [0.59, 2.63], *p* = 0.56, adjusted *p* = 0.65).

**Figure 4:**
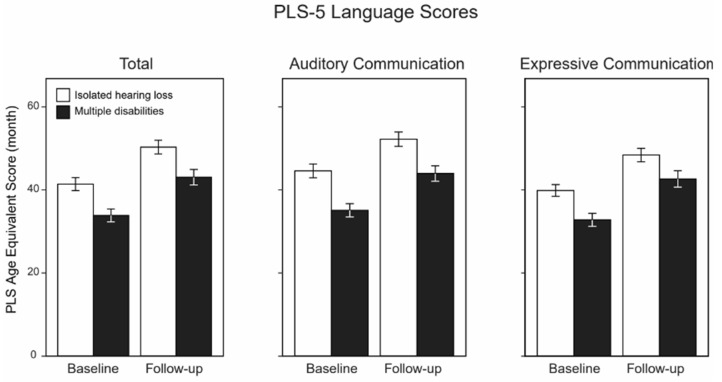
Age-equivalent language outcomes (mean ± SEM, in ms). Greater values indicate a higher age equivalent language age in months. There were 29 children with isolated hearing loss who completed PLS-5 language testing, while 36 children with multiple disabilities completed PLS-5 language testing. At follow-up, 28 children with isolated hearing loss completed language testing, and 33 children with multiple disabilities completed language testing.

Figure 4 shows the PLS-5 language scores of all of the children (*n =* 65). No significant differences between the groups existed in the PLS-5 total language score at baseline (t(63) = 1.51, *p* = 0.14, adjusted *p* = 0.27) or follow-up (t(59) = 1.80, *p* = 0.08, adjusted *p* = 0.25). For the PLS-5 AC score, there were also no significant differences at baseline (t(63) = 1.73, *p* = 0.09, adjusted *p* = 0.25) or at follow-up (t(59) = 1.49, *p* = 0.14, adjusted *p* = 0.27), nor for the PLS-5 EC score at baseline (t(63) = 1.62, *p* = 0.11, adjusted *p* = 0.25) or follow-up (t(54.47) = 1.66, *p* = 0.10, adjusted *p* = 0.25). In children who participated in both the baseline and follow-up language assessments, the three baseline and follow-up language scores (total, auditory comprehension, and expressive comprehension) differed in a statistically significant way in both groups (for each of the three language scores separately: *p* and adjusted *p* < 0.001 for children who had only hearing loss, and *p* and adjusted *p* < 0.001 for children with multiple disabilities, paired *t*-test).

Furthermore, in examining the change in PLS-5 total language scores from baseline to follow-up divided by the change in age from baseline to follow-up (the rate), there were no significant differences between the groups (t(59) = 1.09, *p* = 0.28, adjusted *p* = 0.41). Also, no significant differences were noted in the change in PLS-5 AC score from baseline to follow-up (t(59) = 0.89, *p* = 0.38, adjusted *p* = 0.50), and the change in PLS-5 EC score from baseline to follow-up (t(59) = 1.20, *p* = 0.23, adjusted *p* = 0.37). Specifically, the changes in language scores were larger than the follow-up period of about six months: for children with isolated hearing loss, the average change in the PLS-5 total language score was 10.68 ± 1.26 months (mean ± SEM), while the average change in score for children with multiple disabilities was 8.22 ± 0.91 months. The average change in the PLS-5 AC scores for isolated hearing loss control was 9.76 ± 1.28 months, while the average change for children with multiple disabilities was 7.86 ± 0.99 months. In examining the PLS-5 EC score, the average change for the children with isolated hearing loss was 10.16 ± 1.43 months, while the average change for children with multiple disabilities was 8.92 ± 1.26 months (refer to Figure 4).

### 3.4. Regressions between P1 Latency and PLS-5

Table 2 displays 18 correlation coefficients calculated between the P1 latencies and different language outcome scores at baseline and at follow-up. The correlations were calculated for all children with complete datasets and identifiable P1 latencies, and separately for individuals with abnormal and normal P1 latencies. The rationale behind this division was that normal and abnormal may behave in very different ways, e.g., the distribution of abnormal P1 latencies can be much wider. Since the preceding analyses found no differences, either in the trajectories of the P1 latencies or in the proportions of the abnormal P1 latencies between the groups, the correlations were not calculated separately for the isolated hearing loss group and multiple disabilities group. Children with normal P1 latencies at baseline (*n =* 11) and children with abnormal P1 latencies at follow-up (*n =* 9) were excluded from the analyses because, as expected, these groups were too small for statistical analyses. Hearing loss is known to cause abnormally long P1 latencies; on the other hand, adequate hearing rehabilitation shortens and normalizes the P1 latencies. Also, the correlations between the P1 latencies at follow-up and the PLS-5 scores at baseline (top right in the Table 2) were excluded from the analyses because, based on the earlier studies, our hypothesis was that normal P1 responses are a prerequisite for normal language development, and not vice versa.

For all 18 linear regressions, the calculated correlation coefficients were negative. The pure mathematical probability that 18 random numbers would all be negative by chance is extremely low, 1/(2^18^) = 0.0000038 = 0.00038%. When examining the single correlations separately from each other, without multiple comparison corrections, eight of them had *p*-values that were lower than 0.05 (illustrated with asterisks). After multiple comparison correction, three correlations continued to demonstrate statistical significance (shown with gray areas); that is, individuals with normal P1 latencies at follow-up exhibited significant moderate negative correlations with the PLS-5 auditory comprehension scores (r(31) = −0.62, *p* < 0.001, adjusted *p* = 0.001), expressive communication scores (r(31) = −0.61, *p* < 0.001, adjusted *p* = 0.001), and total language scores (r(31) = −0.64, *p* < 0.001, adjusted *p* < 0.001) at follow-up, meaning that the shorter and thus, the more normal the latencies were, the better the language outcomes were. In addition, the correlation between the P1 latencies at follow-up (all children) and the language scores at follow-up showed a significant negative correlation or trend towards a negative correlation (auditory comprehension scores: r(42) = −0.38, *p* = 0.01, adjusted *p* = 0.06; expressive communication scores: r(42) = −0.39, *p* = 0.009, adjusted *p* = 0.05; total language scores: r(42) = −0.43, *p* = 0.004, adjusted *p* = 0.02).

## 4. Discussion

The main findings of this study are that P1 testing is feasible in hearing-impaired children with additional disabilities, and their auditory cortical maturation (as measured by P1 biomarker latencies) and language development (as measured by PLS-5 scores) do not differ from those of children who have only hearing loss—if they receive similar hearing rehabilitation and speech and language therapy. Furthermore, in most of the children, regardless of whether they had other disabilities or not, their P1 latencies decreased, and their language scores improved during the six-month intensive rehabilitation period. The proportions of children who showed a change from abnormal to normal P1 latency classifications during follow-up were also equivalent in both groups. Finally, P1 latencies were negatively correlated with language development at follow-up, meaning that longer P1 latencies were associated with worse language outcomes, and shorter P1 latencies were associated with better language development.

The shortening of the latencies of the cortical P1 responses is a normal age-related phenomenon, which reflects the maturation of cortical auditory processing [44,45]. Consequently, P1 latency can be used to assess auditory cortical maturation [18]. Cortical maturation is dependent on both environmental factors [46] and external inputs during childhood, and auditory deprivation, i.e., the lack of normal sound input, delays the normal age-related shortening of the P1 latencies [18]. Fortunately, hearing restoration can speed up the maturation or even normalize auditory cortical processing. In a previous study of 23 congenitally deaf children fitted with CIs, the P1 latencies shortened rapidly after implantation [47]. Furthermore, in a case study [48], an 11-month-old child showed a P1 latency decrease from abnormal to normal following bilateral hearing aid use. During follow-up, the child continued to display normal P1 responses and good clinical follow-up. However, in another child using bilateral hearing aids, the P1 latencies were abnormally long, which corresponded with poor clinical outcomes. Based on the P1 and other audiologic findings, this child received a CI at 25 months, and the P1 latency normalized within 3 months of CI use [48]. Despite these favorable results regarding the usefulness of P1 latency as an objective biomarker, no previous comprehensive reports exist about the use of P1 biomarkers in children who have disabilities in addition to hearing loss. Only one case study, by Sharma et al. [17], described the usefulness of the P1 biomarker in three children with multiple disabilities (described in the Introduction).

The improvement and normalization of P1 latencies during this study indicates that adequate hearing rehabilitation combined with intensive speech and language therapy provided to the children resulted in improved cortical auditory processing. On the other hand, abnormally long P1 latencies at baseline suggest that maturation was delayed, despite the former rehabilitation. Based on these data, however, it is not possible to separately evaluate the impact of six months of extra language therapy and the impact of 6 months’ longer use of hearing aids and cochlear implants. However, it should be noted that the children had used their hearing aids or CIs for significant durations prior to testing (see Table 1), which suggests that the impact of extra therapy more likely provided the additional benefit. In any case, the current and the previous studies suggest that persistently abnormally long P1 latencies are a sign of inadequate auditory input, and in children who show delayed latencies, increasing hearing aid amplification, extra language therapy, or CI candidacy may be considered. Overall, the P1 biomarker seems to be a practical objective tool that can be added to the standard audiological test battery to better evaluate hearing and benefit the rehabilitation of children with multiple disabilities.

Similarly to the P1 biomarker, the results of this study demonstrated no differences in the PLS-5 total language scores, PLS-5 auditory comprehension scores, or PLS-5 expressive communication scores between the children who had only hearing loss and the children who had hearing loss and additional disabilities. A previous study in pediatric (2.3–14.6 years old) CI users with multiple disabilities found that the children with Usher syndrome auditory and language skills (measured using the meaningful auditory integration scale, MAIS, and the meaningful use of speech scale, MUSS) that were similar to those of children who had no additional disabilities; meanwhile, children with global developmental delay, attention-deficit hyperactivity disorder, or autism spectrum disorder experienced worse auditory skills [23]. Similar results have been found in 5-year-old children with hearing loss and additional disabilities: the language outcomes were better in children with higher cognitive ability [5]. Furthermore, in a study [25] of 49 children with CIs and additional disabilities (developmental delay, speech and language communication needs, autism spectrum disorders, visual impairments, physical needs, or learning difficulties) compared to 221 CI using children without additional disabilities, no significant difference in speech intelligibility existed between the groups at baseline. However, children with additional disabilities continued to show lower auditory and speech outcomes as well as less change from additional listening therapy in comparison to children who had only hearing loss. Also, in 40 cochlear-implanted children with additional disabilities (45% of them having global developmental delay after Rubella infection), language development improved clearly within one year, but less than in 40 children without additional disabilities [24]. Our good language developmental outcomes (i.e., no difference for children without additional disabilities) may have resulted from the intensive extra language therapy for a period of six months, and/or the fact that our study did not include children with any severe neurological or psychiatric disabilities. The present study, while anecdotal, appears to show that children with multiple disabilities made approximately 8–9 months of progress in language outcomes with an average of 6.9 months of additional therapy.

Altogether, the present and previous studies about language development suggest that in a broad spectrum of hearing-impaired children, additional disabilities do not cause extra delays in language skills if the hearing rehabilitation and language therapy are sufficiently intensive, and if there are no severe neurological and psychiatric disabilities present. However, certain types of additional disabilities, e.g., Down syndrome [49], most likely affect the expected outcomes of language development, whereas in other disabilities such as autism spectrum disorder, the language outcomes may show considerable individual variability, from very good to quite low results [50]. Therefore, future research should examine in more detail language development and the benefits of additional language therapy, as well as dosing effects of hearing rehabilitation in children with different types of additional disabilities. Furthermore, half of the children in this study received their additional therapy via telehealth. A recent review found that family satisfaction with telehealth was high for children with multiple disabilities [51], and thus future research should continue to explore the use of telehealth in this population.

In our regression analyses, all of the calculated 18 correlations resulted in a negative correlation coefficient value. While only three of these correlations (normal P1 latencies at follow-up with the PLS-5 total language scores, PLS-5 auditory comprehension scores, and PLS-5 expressive communication scores) remained significant after correcting *p*-values for multiple comparisons, we observed a clear trend of P1 latency decreases being associated with language increases. Such a negative correlation between cortical development and language development is meaningful, in that decreasing P1 latency reflects an auditory system that is more mature and more efficient in auditory processing, and thus is related to greater language development.

Overall, these results indicate that P1 latencies are associated with language development in hearing-impaired children, regardless of whether they have other disabilities or not. Although we did not find statistically significant correlations between the baseline P1 latencies and later language development, abnormally long P1 latencies most probably imply poor language development. This is consistent with previous studies of P1 latency development and language development in children with CIs [52,53,54,55]. Also, the negative deflection following the P1 response has been suggested to be sensitive to future language outcomes [56,57,58]. For children with multiple disabilities in whom reliable behavioral responses may be difficult to obtain, an objective biomarker that predicts language acquisition could be very useful in directing clinical therapies and education, and in counseling parents on the expectations from hearing rehabilitation [48,52]. Future research may wish to examine if the P1 biomarker may relate to language outcomes differently in some specific disorders, by studying children with and without hearing loss. Additionally, future research could explore in more detail which aspects of language are mostly reflected by the P1 biomarker.

### Limitations of the Study

Some limitations of this study include the small sample size and the retrospective design. In addition to incomplete datasets of some children, we did not have access to all medical records such as audiograms and detailed diagnostic criteria used for various disabilities. However, the diagnoses were made using standard clinical practice, and our aim was not to study specific syndromes or disabilities, but to study a broad spectrum of hearing-impaired children. Our heterogenic sample is a good representation of the real-life patient population in pediatric audiology units. The etiology of the hearing loss varied from congenital to acquired, and for many children, the relationship between the hearing loss and additional disabilities was unclear. Thus, this study provides relevant information on a clinically important everyday topic. However, because our study sample did not include children with the most difficult neurological or psychiatric symptoms, the results of P1 latencies and language development cannot be extended to those cases. However, our results suggest that P1 testing may be possible for severely disabled children, and it is worth trying. Although a large number of hearing-impaired children have various additional disabilities and, for example, the number of different syndromes is large, data about auditory cortical maturation in these children are extremely scarce. The present study provides a comprehensive overview about cortical and language development in children with multiple disabilities. Future detailed prospective studies of different syndromes and clinical entities, such as severe autism spectrum disorder or severe-to-profound intellectual disability, are warranted.

## 5. Conclusions

In a wide spectrum of hearing-impaired children with additional disabilities, auditory cortical maturation (as measured by P1 latencies) and language development (as measured by PLS-5 scores) are similar to children who have only hearing loss, as long as strong hearing rehabilitation is provided. Furthermore, P1 latencies show meaningful correlations with language development. The P1 biomarker is a useful objective tool for physicians, audiologists, and other clinicians to evaluate the development of the auditory cortex and the sufficiency of hearing rehabilitation in children with multiple disabilities, whose disabilities may prevent reliable behavioral responses. The EEG-based P1 biomarker is easy to measure, demonstrates good replicability, is rather inexpensive, and is suitable both in hearing aid and CI users. Information about auditory cortical maturation, and thus about the benefits of current hearing rehabilitation, can help in clinical decision-making, such as switching from hearing aids to CIs.

## Figures and Tables

**Table 1:** Summary of the participants.

		All Children ^1^		Complete Dataset	
		Multiple Disabilities	Isolated Hearing Loss	Multiple Disabilities	Isolated Hearing Loss
**Number of the patients**		36	26	22	23
**Demographics**	Age (mos; mean ± SD)	41.3 ± 17.6	48 ± 20.0	46.1 ± 16.8	49.9 ± 19.6
	Gender (% of females)	56.4	53.8	50	47.8
	ASL (% of ASL Users)	16.7	30.8	22.7	34.8
**Hearing rehabilitation**	Hearing aid (%)	33.3	46.2	45.5	47.8
	Cochlear implant (%) ^2^	66.7	53.8	54.5	52.2
	Age of Amplification (mos; mean ± SD) ^3^	15.1 ± 17.1	9.35 ± 13.21	19.18 ± 18.75	10.30 ± 13.78
	Age of implantation (mos; mean ± SD) ^4^	24.3 ± 17.5	18.4 ± 13.3	32.2 ± 20.0	19.4 ± 14.2
	Duration of Hearing Rehabilitation	26.28 ± 19.1	38.69 ± 21.07	19.18 ± 20.32	10.30 ± 21.41
**Speech therapy**	In person	52.8	50.0	54.5	52.2
	Telehealth	38.9	42.3	40.9	39.1
	Length (mos; mean ± SD)	6.9 ± 1.4	7.6 ± 2.5	6.9 ± 1.4	7.6 ± 2.6

^1^ includes children with incomplete dataset, ^2^ bilateral, unilateral, and bimodal users, ^3^ age of first hearing aid or cochlear implant, ^4^ age of first cochlear implant.

**Table 2:** Pearson correlation coefficients between P1 latencies and language outcomes.

			P1 Latency					
			Baseline			Follow-Up		
			All Children	Abnormal P1	Normal P1	All Children	Abnormal P1	Normal P1
	Baseline	PLS-5 Total	−0.25	−0.34	SUP	N/A		
		PLS-5 AC	−0.24	−0.33	SUP			
**Language**		PLS-5 EC	−0.28	−0.34	SUP			
**score**	Follow-up	PLS-5 Total	−0.33 *	−0.43 *	SUP	−0.43 ***	SUP	**−** 0.64 ***
		PLS-5 AC	−0.25	−0.41 ~	SUP	−0.38 *	SUP	**−** 0.62 ***
		PLS-5 EC	−0.29	−0.37	SUP	−0.39 **	SUP	**−** 0.61 ***

Uncorrected *p*-values: *** *p* < 0.005, ** *p* < 0.01, * *p* < 0.05, ~ *p* < 0.07. N/A = not applicable, SUP = statistically unpowered sub-group.

## Data Availability

The data used in this study are available from the corresponding author upon reasonable request.

## Supplementary Materials

The following supporting information can be downloaded at: https://www.mdpi.com/article/10.3390/children10111813/s1.

## References

[1] Tekin M., Arnos K.S., Pandya A. (2001). Advances in hereditary deafness. Lancet.

[2] Finsterer J., Fellinger J. (2005). Nuclear and mitochondrial genes mutated in nonsyndromic impaired hearing. Int. J. Pediatr. Otorhinolaryngol..

[3] Home—OMIM. https://www.omim.org/.

[4] Shearer A.E., Hildebrand M.S., Schaefer A.M., Smith R.J., Adam M.P., Mirzaa G.M., Pagon R.A., Wallace S.E., Bean L.J., Gripp K.W., Amemiya A. (1993). Genetic Hearing Loss Overview. GeneReviews^®^.

[5] Cupples L., Ching T.Y.C., Button L., Leigh G., Marnane V., Whitfield J., Gunnourie M., Martin L. (2018). Language and speech outcomes of children with hearing loss and additional disabilities: Identifying the variables that influence performance at five years of age. Int. J. Audiol..

[6] Picard M. (2004). Children with permanent hearing loss and associated disabilities: Revisiting current epidemiological data and causes of deafness. Volta. Rev..

[7] Birman C.S., Elliott E.J., Gibson W.P.R. (2012). Pediatric cochlear implants: Additional disabilities prevalence, risk factors, and effect on language outcomes. Otol. Neurotol..

[8] Johnson K.C., Wiley S. (2009). Cochlear Implantation in Children with Multiple Disabilties. Clinical Management of Children with Cochlear Implants.

[9] Guidelines for the Early Audiological Assessment and Management of Babies Referred from the Newborn Hearing Screening Programme. British Society of Audiology. https://www.thebsa.org.uk/resources/.

[10] Clinical Guidance Document Assessment of Hearing in Infants and Young Children. American Academy of Audiology. https://www.audiology.org/wp-content/uploads/2021/05/Clin-Guid-Doc_Assess_Hear_Infants_Children_1.23.20.pdf.

[11] Gorga M.P., Johnson T.A., Kaminski J.R., Beauchaine K.L., Garner C.A., Neely S.T. (2006). Using a combination of click- and tone burst–evoked auditory brain stem response measurements to estimate pure-tone thresholds. Ear Hear..

[12] Cohen I.L., Gardner J.M., Karmel B.Z., Phan H.T.T., Kittler P., Gomez T.R., Gonzalez M.G., Lennon E.M., Parab S., Barone A. (2013). Neonatal brainstem function and 4-month arousal-modulated attention are jointly associated with autism. Autism Res..

[13] Delgado C.F., Simpson E.A., Zeng G., Delgado R.E., Miron O. (2023). Newborn auditory brainstem responses in children with developmental disabilities. J. Autism Dev. Disord..

[14] Childhood Hearing Screening American Speech-Language-Hearing Association. https://www.asha.org/practice-portal/professional-issues/childhood-hearing-screening.

[15] Aldè M., Di Berardino F., Ambrosetti U., Barozzi S., Piatti G., Consonni D., Zanetti D., Pignataro L., Cantarella G. (2022). Hearing outcomes in preterm infants with confirmed hearing loss. Int. J. Pediatr. Otorhinolaryngol..

[16] Corrales C.E., Oghalai J.S. (2013). Cochlear implant considerations in children with additional disabilities. Curr. Otorhinolaryngol. Rep..

[17] Sharma A., Glick H., Campbell J., Biever A. (2013). Central auditory development in children with hearing loss: Clinical relevance of the P1 CAEP biomarker in hearing-impaired children with multiple disabilities. Hear. Balance Commun..

[18] Sharma A., Dorman M.F., Spahr A.J. (2002). A sensitive period for the development of the central auditory system in children with cochlear implants: Implications for age of implantation. Ear Hear..

[19] Dorman M.F., Sharma A., Gilley P., Martin K., Roland P. (2007). Central auditory development: Evidence from CAEP measurements in children fit with cochlear implants. J. Commun. Disord..

[20] Sharma A., Dorman M., Spahr A., Todd N.W. (2002). Early cochlear implantation in children allows normal development of central auditory pathways. Ann. Otol. Rhinol. Laryngol. Suppl..

[21] Sharma A., Gilley P.M., Dorman M.F., Baldwin R. (2007). Deprivation-induced cortical reorganization in children with cochlear implants. Int. J. Audiol..

[22] Broomfield S.J., Bruce I.A., Henderson L., Ramsden R.T., Green K.M.J. (2013). Cochlear implantation in children with syndromic deafness. Int. J. Pediatr. Otorhinolaryngol..

[23] Mesallam T.A., Yousef M., Almasaad A. (2019). Auditory and language skills development after cochlear implantation in children with multiple disabilities. Eur. Arch. Oto-Rhino-Laryngol..

[24] Ganesh V., Ram B., Nandhan R., Kameswaran M. (2021). A retrospective clinical audit of outcomes of cochlear implantation in children with multiple disabilities in comparison with normal implantees: A south Indian experience. Indian J. Otolaryngol. Head. Neck Surg..

[25] Rawes C., Ngaage L.M., Mackenzie R., Martin J., Cordingley A., Raine C. (2021). A review of the outcomes of children with designated additional needs receiving cochlear implantation for severe to profound hearing loss. Cochlear Implants Int..

[26] Eggermont J.J. (1988). On the rate of maturation of sensory evoked potentials. Electroencephalogr. Clin. Neurophysiol..

[27] Wunderlich J.L., Cone-Wesson B.K., Shepherd R. (2006). Maturation of the cortical auditory evoked potential in infants and young children. Hear. Res..

[28] Ponton C.W., Eggermont J.J., Kwong B., Don M. (2000). Maturation of human central auditory system activity: Evidence from multi-channel evoked potentials. Clin. Neurophysiol..

[29] Pang E.W., Taylor M.J. (2000). Tracking the development of the N1 from age 3 to adulthood: An examination of speech and non-speech stimuli. Clin. Neurophysiol..

[30] Sharma A., Kraus N., McGee T.J., Nicol T.G. (1997). Developmental changes in P1 and N1 central auditory responses elicited by consonant-vowel syllables. Electroencephalogr. Clin. Neurophysiol..

[31] Eggermont J.J., Ponton C.W. (2003). Auditory-evoked potential studies of cortical maturation in normal hearing and implanted children: Correlations with changes in structure and speech perception. Acta Otolaryngol..

[32] 2012 Audiologic Guidelines for the Assessment of Hearing in Infants and Young Children. American Academy of Audiology. https://www.audiology.org/practice-guideline/2012-audiologic-guidelines-for-the-assessment-of-hearing-in-infants-and-young-children/.

[33] Gilley P.M., Sharma A., Dorman M., Finley C.C., Panch A.S., Martin K. (2006). Minimization of cochlear implant stimulus artifact in cortical auditory evoked potentials. Clin. Neurophysiol..

[34] Sahli A.S., Belgin E. (2017). Adaptation, validity, and reliability of the Preschool Language Scale-Fifth Edition (PLS-5) in the Turkish context: The Turkish Preschool Language Scale-5 (TPLS-5). Int. J. Pediatr. Otorhinolaryngol..

[35] Zimmerman I.L., Steiner V.G., Pond R.E. (2012). Preschool Language Scale, Fifth Edition. http://doi.apa.org/getdoi.cfm?doi=10.1037/t15141-000.

[36] R Core Team (2022). R: A Language and Environment for Statistical Computing. Vienna, Austria: R Foundation for Statistical Computing. https://www.R-project.org/.

[37] R Studio Team (2022). RStudio: Integrated Development Environment for R. Boston, MA: RStudio, PBC..

[38] Bates D., Mächler M., Bolker B., Walker S. (2015). Fitting Linear Mixed-Effects Models Using lme4. J. Stat. Softw..

[39] Schielzeth H., Dingemanse N.J., Nakagawa S., Westneat D.F., Allegue H., Teplitsky C., Réale D., Dochtermann N.A., Garamszegi L.Z., Araya-Ajoy Y.G. (2020). Robustness of linear mixed-effects models to violations of distributional assumptions. Methods Ecol. Evol..

[40] le Cessie S., Goeman J.J., Dekkers O.M. (2020). Who is afraid of non-normal data? Choosing between parametric and non-parametric tests. Eur. J. Endocrinol..

[41] Skovlund E., Fenstad G.U. (2001). Should we always choose a nonparametric test when comparing two apparently nonnormal distributions?. J. Clin. Epidemiol..

[42] Fagerland M.W. (2012). T-tests, non-parametric tests, and large studies--a paradox of statistical practice?. BMC Med. Res. Methodol..

[43] Benjamini Y., Hochberg Y. (1995). Controlling the false discovery rate: A practical and powerful approach to multiple testing. J. R. Stat. Soc. Ser. B Methodol..

[44] Wunderlich J.L., Cone-Wesson B.K. (2006). Maturation of CAEP in infants and children: A review. Hear. Res..

[45] Moore J.K., Linthicum F.H. (2007). The human auditory system: A timeline of development. Int. J. Audiol..

[46] Tooley U.A., Bassett D.S., Mackey A.P. (2021). Environmental influences on the pace of brain development. Nat. Rev. Neurosci..

[47] Sharma A., Dorman M.F., Kral A. (2005). The influence of a sensitive period on central auditory development in children with unilateral and bilateral cochlear implants. Hear. Res..

[48] Sharma A., Martin K., Roland P., Bauer P., Sweeney M.H., Gilley P., Dorman M. (2005). P1 latency as a biomarker for central auditory development in children with hearing impairment. J. Am. Acad. Audiol..

[49] Clarós P., Remjasz A., Clarós-Pujol A., Pujol C., Clarós A., Wiatrow A. (2019). Long-term outcomes in down syndrome children after cochlear implantation: Particular issues and considerations. Otol. Neurotol..

[50] Eshraghi A.A., Nazarian R., Telischi F.F., Martinez D., Hodges A., Velandia S., Cejas-Cruz I., Balkany T.J., Lo K., Lang D. (2015). Cochlear implantation in children with autism spectrum disorder. Otol. Neurotol..

[51] Ferro F., Tozzi A.E., Erba I., Dall’Oglio I., Campana A., Cecchetti C., Geremia C., Rega M.L., Tontini G., Tiozzo E. (2021). Impact of telemedicine on health outcomes in children with medical complexity: An integrative review. Eur. J. Pediatr..

[52] Cardon G., Sharma A. (2013). Central auditory maturation and behavioral outcome in children with auditory neuropathy spectrum disorder who use cochlear implants. Int. J. Audiol..

[53] Lee S.Y., Han J.H., Song H.K., Kim N.J., Yi N., Kyong J.S., Choi B.Y. (2021). Central auditory maturation and behavioral outcomes after cochlear implantation in prelingual auditory neuropathy spectrum disorder related to OTOF variants (DFNB9): Lessons from pilot study. PLoS ONE.

[54] Silva L.A.F., Couto M.I.V., Magliaro F.C.L., Tsuji R.K., Bento R.F., de Carvalho A.C.M., Matas C.G. (2017). Cortical maturation in children with cochlear implants: Correlation between electrophysiological and behavioral measurement. PLoS ONE.

[55] Cavalcanti M.I., Silva L.A.F., Goffi Gomez M.V.S., Koji T.R., Bento R.F., Martinho de Carvalho A.C., Gentile M.C. (2021). Central auditory nervous system stimulation through the cochlear implant use and its behavioral impacts: A longitudinal study of case series. Case Rep. Otolaryngol..

[56] Benasich A.A., Choudhury N., Friedman J.T., Realpe-Bonilla T., Chojnowska C., Gou Z. (2006). The infant as a prelinguistic model for language learning impairments: Predicting from event-related potentials to behavior. Neuropsychologia.

[57] Choudhury N., Benasich A.A. (2011). Maturation of auditory evoked potentials from 6 to 48 months: Prediction to 3 and 4 year language and cognitive abilities. Clin. Neurophysiol..

[58] Choudhury N., Leppanen P.H.T., Leevers H.J., Benasich A.A. (2007). Infant information processing and family history of specific language impairment: Converging evidence for RAP deficits from two paradigms. Dev. Sci..

