# Supplementary material for: Auditory Cortex Maturation and Language Development in Children with Hearing Loss and Additional Disabilities
Source: Children (Basel). 2023 Nov 15;10(11):1813. doi: 10.3390/children10111813 (PMC10670362; doi:10.3390/children10111813)
Supplement: Supplementary file 1 [file children-10-01813-s001.zip › children-2621257-supplementary.pdf]

Disabilitiy	*n*
Charge	1
Cleft Palate	1
CMV	2
CMV and Fine Motor	1
CMV and Gross Motor	2
CMV, Fine and Gross Motor	2
Epilepsy	1
Epilespy, Fine and Gross Motor	1
Fine and Gross Motor	1
Fine Motor	3
Fine Motor and Epilepsy	1
Gross Motor	2
Gross Motor and Hypotonia	1
Heart Defect	3
Heart Defect and Asthma	1
Hypotonia, Asthma, and Dysphagia	1
Meningitis	1
Metabolic Disorder	1
Missing Kidney	1
Pendred	2
Traumatic Brain Injury	1
Usher Type II	1
Waardenburg	4
Waardenburg and Gross Motor	1
